# Binocular non-stereoscopic cues can deceive clinical tests of stereopsis

**DOI:** 10.1038/s41598-019-42149-2

**Published:** 2019-04-08

**Authors:** Adrien Chopin, Samantha Wenyan Chan, Bahia Guellai, Daphné Bavelier, Dennis Michael Levi

**Affiliations:** 1 Department of Psychology and Education Sciences, University of Geneva, Geneva, Switzerland; 2 School of Optometry, University of California, Berkeley, USA; 3 Laboratoire Ethologie, Cognition, Développement, Université Paris Nanterre, Nanterre, France

## Abstract

Stereoscopic vision plays a critical role in visual perception; however, it is difficult to assess. In clinical settings, stereoacuity is assessed with clinical stereotests. Observers can use monocular cues to deceive some of the most common stereotests, such as the Titmus test. The Randot test has been found free of monocular cues, and here we confirm that result by testing observers under monocular viewing. However, there is a common misconception that only monocular cues can be used to deceive stereotests. Here we demonstrate that binocular non-stereoscopic cues can also be used to pass the Randot, by testing participants with the test rotated, a condition that abolishes stereopsis, and comparing the performance to a monocular viewing condition. We also assessed the Random Dot Butterfly test and discovered considerable amounts of non-stereoscopic cues, including binocular cues in the Circles that can be used to deceive the test. Participants with amblyopia had more difficulty using non-stereoscopic cues than neurotypical observers. We gathered normal-viewing Randot stereoacuities for 110 participants (90 neurotypical and 20 with amblyopia) and compared them to psychophysical stereoacuities (our gold standard). The Randot test showed low positive normalized predictive values for detecting stereoblindness. It could perfectly detect stereo-impairment but with a low sensitivity.

## Introduction

One important aspect of clinical assessment of visual function is the assessment of binocular vision^1^. It is routinely assessed with clinical stereotests that measure stereoscopic vision^2^ – the ability to perceive depth from the difference of viewpoints between the two eyes.

Stereopsis is functionally important for vision-related quality of life^3^, object prehension^4^, object placement^5^, rapid distance estimation^6,7^ and for other tasks involving fine hand movements^8^. Around 5% of people^9,10^ are reported to be impaired by a total lack of stereoscopic vision (stereoblindness) and a larger proportion have difficulty using stereoscopic cues for depth perception^3^ (stereo-impairment), perhaps as high as 30 percent^11^. Stereo-impairments are often associated with widespread binocular anomalies like amblyopia^5,12^, anisometropia or strabismus^13^. As a result, stereoacuity measurement is the clinical standard for diagnosing the presence and quality of binocular vision^1^. Stereoacuity is also commonly used to assess the quality of binocular visual function in experimental settings.

Yet, stereoacuity measurements are usually collected through clinical stereotests, often with mixed success in identifying binocular anomalies^14^. This poor diagnostic efficacy could be related to the fact that accurately measuring stereoacuity is challenging. The test-retest reliability of many stereotests is poor: results vary by as much as a factor of four^15^. The reason for this variability may be related to the presence of several non-stereoscopic cues to depth in the tests: (i) monocular cues and (ii) binocular non-stereoscopic cues. Those cues first need to be removed from the display before the genuine stereoscopic depth performance can be reliably and validly assessed. Monocular cues include perspective, motion parallax^16^, the alternating fixation described by Archer^17^ and more generally any aspect of the display that would be perceptible with only one eye. The availability of these cues to pass clinical stereotests has been clearly demonstrated in the Titmus Fly and Titmus Circles (Stereo Optical Co., Chicago, IL, USA)^18–22^, the Random Dot E (Stereo Optical Co.)^23^ or the Frisby-Davis 2 (Clement Clarke International, Harlow, UK)^24^. Evidence for monocular cues was provided by showing that the test could be passed monocularly or by stereoblind persons more often than by chance.

However, in addition to monocular cues, there may also be binocular non-stereoscopic cues. Cooper^25^ noted that the cues are probably present in random-dot stereograms: he termed them as *decorrelation cues*. The possibility that these binocular non-stereoscopic cues might be used by patients who lack genuine stereopsis to correctly report the shapes in clinical random-dot stereograms was also noted by Charman and Jennings^26^. They observed that shapes in the random-dot stereograms of the TNO could be identified when the test was rotated 90 degrees so that horizontal disparities become vertical. Only horizontal disparities give rise to stereopsis, so that stereoscopic depth disappears in rotated viewing. This is why the rotated viewing was prescribed in clinical setting as a safety net^27^. Rotated viewing has been used in developmental studies in both humans^28^ and monkeys^29^; however, it has not been widely adopted in clinical studies.

Charman and Jennings’ interpretation was that the incompatible images triggered binocular rivalry, a noticeable alternation of each eye’s image, while the background could still be fused. They reasoned that stereoblind participants could also use binocular rivalry to identify shapes in stereograms when the test is not rotated. In other words, random-dot stereograms introduce effective binocular non-stereoscopic cues to pass the test. No empirical evidence was provided to substantiate any of these claims. It is possible that stereoblind patients who notice the shape in rivalry would ignore it because it does not appear in depth. However, it is likely that the rivalrous area would genuinely appear to float in depth behind the fused area, a phenomenon fully documented and coined *rivaldepth*^30^. An alternative possibility is that binocular non-stereoscopic cues do not necessarily manifest as alternations as in binocular rivalry but can simply appear as a binocular luster^31^ or as a diplopic or confusion area^32^. A last cue worth considering is delta-vergence. It is the strategy of alternating eye fixations between the different depth planes of two visual objects, even in the absence of stereoscopic depth perception. It is possible because the vergence system can use absolute disparities unlike the perceptual depth system, which is mostly blind to them^34,35^. Thus using delta vergence, the observer may monitor the vergence changes and deduce from these the depth ordering. It will not be discussed further as it is a depth cue that unlike the ones discussed above can be classified as stereoscopic^33^.

In summary, non-stereoscopic cues encompass monocular cues and at least four potential binocular non-stereoscopic cues: binocular luster, diplopia/confusion, binocular rivalry, and rivaldepth (Table 1). In theory, any or all of these cues could be used to identify shapes or items in depth in random dot stereograms in the total absence of stereoscopic vision.

**Table 1:** The different cues to pass stereotests.

Non-stereoscopic cues		Stereo cue
Monocular cues	Binocular cues	
Motion parallax*	Binocular rivalry	Relative disparity*
Perspective*	Diplopia/Confusion	Absolute disparity
Shadows*	Binocular luster	Delta-vergence*
Alternating fixation (…)	Rivaldepth	

The table describes the relations between the different categories of cues to pass stereotests. Some are cues to depth and likely to participate in everyday-life depth perception (indicated with an asterisk *).

The present experiment separately evaluates the presence of monocular and binocular non-stereoscopic cues in the two widely used clinical tests described below: the Randot and Random Dot Butterfly stereotests. Both tests are commonly used to assess stereoacuities in research studies.

The Randot test (Stereo Optical Co.)^36^ is a commonly-used vectograph clinical stereotest made of different portions, including a Shape portion (a series of shapes hidden in random-dot stereograms) and a Circles portion (a group of 3–4 Wirt circles with one circle in front of the other). It derived from the Titmus test, with modifications designed to remove monocular cues. The first version contained a random-dot version of the Circles^36^ that was demonstrated to be free of monocular cues^22,37–39^. A later version (modified version 2) used contour-based circles on a random-dot background. Three studies confirmed the absence of monocular cues^21,39,40^ in that version, while another study concluded that participants could detect the circles in depth with the two largest disparity levels monocularly^22^. However, by our calculation, the reported proportions of success were not higher than chance in that study. While monocular cues are absent in the Randot Circles, binocular non-stereoscopic cues might still be present in both portions.

The Random Dot Butterfly stereotest (Stereo Optical Co.) combines the Titmus version of the Circles and a random-dot stereogram depicting a large hidden butterfly. The test has been advocated as an adjunct for screening strabismic patients^41^, was used as a gold standard for stereopsis^42^, recommended for adult screening^43^, and is sometimes used in clinical practice. As mentioned earlier, we already know that the Circles portion of this test contains monocular cues. When 11 patients with binocular dysfunction viewed the Titmus Circles binocularly, their strong suppression created monocular cues visible as a lateral offset^18^. We wondered whether other non-stereoscopic cues were present in the absence of sustained interocular suppression (i.e., in normal vision). In the Shape portion of that test, the presence of binocular non-stereoscopic cues has never been investigated.

In the current study, we evaluate the presence of monocular and binocular non-stereoscopic cues in two commonly used clinical tests (Randot and Butterfly tests). Under 90-degrees-rotated viewing, horizontal disparities become vertical and genuine stereoscopic vision is eliminated, while potential binocular non-stereoscopic cues and monocular cues remain unchanged. For the Randot, we evaluate the exclusive presence of monocular cues with a monocular viewing condition. For the Butterfly Circles, we pooled together data from the abundant literature on that question^18–22,44^. The difference between monocular and rotated viewing shows the extent to which binocular non-stereoscopic cues can be used. In addition, we compare stereoacuities measured with the Randot test to those measured with several psychophysical stereotests that are free of non-stereoscopic cues. Finally, in stereo-training studies, it is often crucial to isolate stereoblind or stereo-impaired observers. Therefore, we assess the Randot’s diagnostic accuracy for stereoblindness and stereo-impairment.

## Methods

We conducted a prospective experimental study (laboratory investigation) in which we compared stereoacuities measured in the same participants with 2 clinical stereotests (Randot and Random Dot Butterfly) in three viewing conditions (monocular, binocular and rotated) and computerized psychophysical stereotests as a measure of “true” stereoacuity. The experiments followed the Declaration of Helsinki, were HIPAA-compliant when conducted with patients in the USA. Approval of the Berkeley Committee for the Protection of Human Subjects and UNIGE’s Ethics Committee was obtained prior to the study and we followed their guidelines. All participants were fully naive to the goals of the experiment (except for the authors), gave their written informed consent and obtained fair monetary compensation for their participation.

### Study population

We tested different samples of adult participants recruited in Geneva and Berkeley (see Supplementary Table S1 for details) for a total of 110 observers. Some of the data has been previously partially reported^34,45^. Ninety had typical vision (defined as a monocular SLOAN corrected acuity better or equal to 20/20) and 20 were amblyopic. Details of recruitment procedures and a further description of each sample are available in the Supplementary Methods online. Clinical details of the amblyopic participants are provided in Supplementary Table S3.

### Observation procedure

#### Measuring stereoacuities with psychophysical tests

We used three different psychophysical tests to measure stereoacuities, but not necessarily on all participants (for most observers only one of the three tests was used). The specific test used for each participant can be found in Supplementary Table S2. The J-RDS test (n = 6) is a computerized test based on dynamic random-dot stereograms. It uses the constant-stimulus method for varying the relative disparity of a central square in the middle of a larger background square. Presentation time was 200 ms. The Eyetracked-RDS (n = 23) is an eyetracked version of the J-RDS, which enabled us to display the stereograms for 2000 ms, while avoiding the delta-vergence strategy. The third psychophysical test (n = 35) is based on the single-stimulus method with lines presented at different disparities. The stimuli and procedures for those tests are fully described in the Supplementary Methods. Participants passed the Diplopia-Suppression test before running the J-RDS and Eyetracked-RDS stereotests, which helps ensure proper fusion and minimal interocular suppression. The stimuli and procedure for the Diplopia-Suppression Test are described in the Supplementary Methods.

#### Randot clinical stereotest

We used the commercial Randot stereotests (modified version, also called version 2) produced by Stereo Optical Co., Inc. The exact detail and order of presentation for each viewing condition and each participant is available in Supplementary Table S2. The Shape-portion of the Randot test was tested rotated first for 84 participants, and then either viewed upside-down (n = 64), right-side up (n = 19), or both (n = 1). Ninety-three participants viewed the Circles-portion of the Randot rotated and right-side up. Forty participants were also tested with upside-down viewing and 28 with monocular (patched) viewing.

#### Butterfly clinical stereotest

The order of presentation for each viewing condition for the Butterfly test is available in the Supplementary Table S2. All participants in samples 1 and 2 (n = 57) were tested with the shape-portion of the Butterfly stereotest viewed rotated first and then right-side up. Fifty-six participants were also tested with the circles-portion viewed right side up, upside down and rotated.

### Analysis

All statistics were calculated using Matlab R2016b and power analyses were post-hoc two-tailed analyses conducted with G*power. A measureable stereoacuity is defined as any non-zero stereoacuity on clinical stereotests, any stereoacuity better than 2000″ for the psychophysical stereotests Eyetracked-RDS and J-RDS and better than the 95^th^ percentile of our sample for the single-stimulus method. All tests are two-tailed and all alpha levels were 5%. Distributions of clinical stereotest scores were not normally distributed (Lilliefors’ test KS = 0.24; p = 0.0048 for Butterfly test, and KS = 0.42; p = 0.001 for Randot test), therefore we used the non-parametric Kruskal-Wallis test to analyze order effects. Distributions of Randot scores were not normally distributed either when separated between amblyopic and neurotypical samples (Lilliefors’ test KS = 0.36; p = 0.001 for the sample with amblyopia, and KS = 0.37; p = 0.001 for the neurotypical sample), therefore we used the non-parametric Mann-Whitney test to compare distributions.

## Results

### Non-stereoscopic cues in the Randot Shapes

#### Patched-viewing (monocular) scores

None of the participants who had measurable stereopsis when tested right side up had measurable stereopsis in the Randot Shapes when viewed with their weaker eye patched (a probability not different from chance; binomial test, p = 0.20; n = 28). Only monocular cues are present when the test is performed under patched conditions. Therefore, monocular cues are absent in the Randot Shapes. Order of presentation was counter-balanced between participants.

#### Rotated-viewing (binocular) scores

Of the 71 participants who had measurable stereopsis with the Randot Shapes viewed right side up or upside-down, 55 (77.4%; CI_0.95_ = [0.68–0.87]) had measurable stereopsis when the test was rotated by 90 degrees. No stereoscopic cues are present in the test when rotated. Participants are not expected to be able to correctly identify the shapes when the test is rotated if non-stereoscopic cues are absent. This means that non-stereoscopic cues are present in this portion of the test. Given that no monocular cues were present, all non-stereoscopic cues were binocular. Interestingly, the thirteen amblyopic patients who had no measurable stereopsis when the Randot Shapes were viewed right side up also had no measurable stereopsis when the test was rotated. This suggests that existing non-stereoscopic cues in the Randot Shapes may be difficult to use by amblyopic patients. Finally, three of eleven participants (27%) who were stereoblind according to psychophysical tests could pass this part of the test in rotated viewing, using binocular non-stereoscopic cues then. Note that observers started with the rotated viewing before right-side up or upside-down viewing, in order not to bias their responses.

#### Relation between scores in rotated viewing and non-rotated viewing

**Figure 1:**
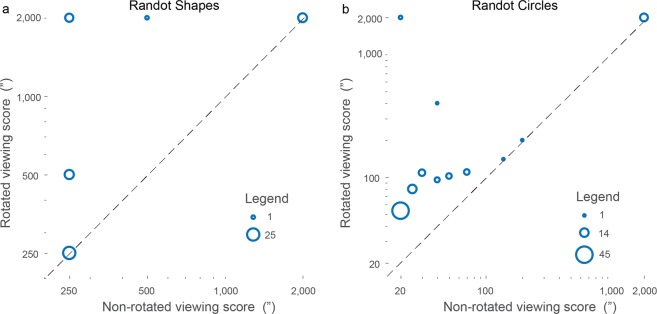
Relation between individual stereotest scores (in arcsec, ”) when the test is viewed rotated vs. non-rotated for the Randot Shapes (**a**) or the Randot Circles (**b**). The non-rotated score averages between scores when the test is viewed right-side up or upside-down whenever more than one score is available. The dashed line is the identity line. The size of the circles is proportional to the number of data in the area. When the ordinate-axis distance between two data points is smaller than 10% of their value, data points are pooled together.

As a confirmatory analysis, we determined whether the participants who were better at using binocular non-stereoscopic cues also scored better under non-rotated viewing. In theory, if binocular non-stereoscopic cues are useful for passing the test under non-rotated viewing, the scores in rotated and non-rotated viewing should be correlated. Indeed, the correlation was moderate to strong (Spearman r = 0.61; p = 8.10^−8^, n = 65, Fig. 1a).

### Non-stereoscopic cues in the Randot Circles

#### Patched-viewing (monocular) scores

**Figure 2:**
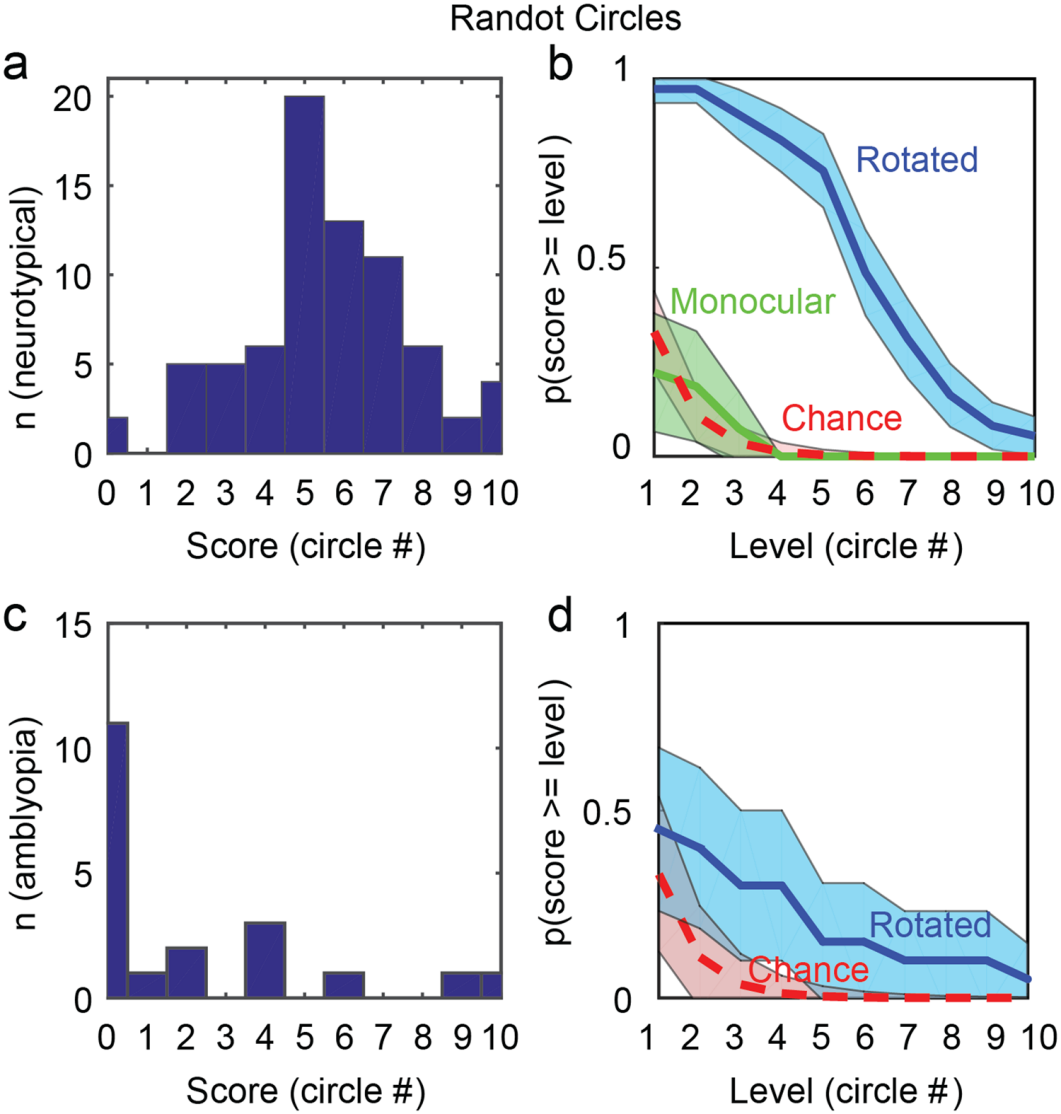
Distribution of scores for the Randot-Circles test, in rotated viewing, for neurotypical (**a**) and amblyopic (**c**) participants. Probability to pass at least the level in abscissa in rotated viewing (blue solid line) and monocular viewing (green solid line) for neurotypical (**b**) and amblyopic (**d**) participants. The probability to pass the level in abscissa by chance is indicated with the red dashed line. Shaded areas depict the 95% confidence interval. Scores/levels for each reached circle: 1: 400″, 2: 200″, 3: 140″, 4: 100″, 5: 70″, 6: 50″, 7: 40″, 8: 30″, 9: 25″, 10: 20″.

When participants viewed the Randot Circles test with their weaker eye patched, the proportion of participants reaching each circle level with monocular viewing was not significantly different from chance (Fig. 2b; n = 27; binomial tests, all levels p > 0.16; 1-β = 1), demonstrating the absence of effective monocular cues to pass the test. Note that circle levels (Fig. 2, abscissa) denote disparities from 400″ (level 1), to 20″ (level 10).

#### Rotated-viewing (binocular) scores

Figure 2 shows the distributions of scores reached with the Circles (a and c) and the probability of reaching each Circle level when observers viewed the test rotated by 90 degrees (b and d). For neurotypical observers, all score proportions differed significantly from what is expected by chance only (Fig. 2b; n = 73; binomial test, p < 10^−14^; 1-β = 1), demonstrating the influence of non-stereoscopic cues at all disparity levels. Seventy-one observers out of 73 (97.3%; CI_0.95_ = [0.94e-1]) could pass at least the second level (200″). The absence of monocular cues demonstrates the presence of useful binocular non-stereoscopic cues to deceive the test.

Importantly, the order of presentation of the rotated viewing (which was counterbalanced between neurotypical participants) did not affect the performance (Kruskal-Wallis Chi square = 3.13, p = 0.21; n = 73).

Similarly, the proportion of amblyopic patients who could identify the correct item when the test was viewed rotated was significantly different from chance for all but the largest disparity (400″; Fig. 2d; n = 20; binomial tests, all level p < 3 × 10^−4^ except for level #1, p = 0.18), demonstrating the influence of non-stereoscopic cues for disparities smaller than 400″. Eight observers (40%; CI_0.95_ = [0.18–0.61]) passed at least the second level (200″). Interestingly, the distributions of rotated-viewing scores for neurotypical (Fig. 2a) and amblyopic participants (Fig. 2c) were significantly different, with the amblyopic observers reaching lower scores (n = 20 and n = 73; Mann-Whitney U = 1432, p = 3.5 × 10^−6^). Again, this suggests that amblyopic patients do not use non-stereoscopic cues as effectively as observers with typical vision. This difference cannot be explained by a difference in order of presentation because amblyopic observers started with right-side-up viewing.

Finally, three of eleven participants (27%) who were stereoblind according to psychophysical tests could pass this part of the test in rotated viewing, confirming the use of binocular non-stereoscopic cues.

#### Relation between scores in rotated viewing and non-rotated viewing

As a confirmatory analysis, we determined whether the participants who were better at using binocular non-stereoscopic cues also scored better under non-rotated viewing. In theory, if binocular non-stereoscopic cues are useful for passing the test under non-rotated viewing, the scores in rotated and non-rotated viewing should be correlated. Indeed, the correlation was moderate to strong (Spearman r = 0.64; p = 5.10^−12^, n = 93, Fig. 1b).

#### Relationship between scores on the Randot Circles and psychophysical stereoacuities

The psychophysical stereotests that we used are immune to binocular non-stereoscopic cues because they rely on signed depth information (is an object in front of or behind another object?) rather than on non-depth information (is an item odd among others? What does the concealed shape look like?). Thresholds in the Randot-Circles test correlated significantly with the best threshold measured with any of our psychophysical stereotest (n = 61; Spearman r = 0.81, p = 1.9 × 10^−7^; 1-β = 1; Supplementary Fig. S2). Therefore, the Randot-Circles test measures something that is closely related to the stereoscopic acuity measured in psychophysical tests, but 34% of the variance (1 − R^2^) could be due to other factors such as the use of binocular non-stereoscopic cues.

#### Can we use the Randot-Circles test to diagnose total stereoblindness

We refer to *true stereoacuity* as the best threshold measured with any of our three psychophysical tests. When the Eyetracked-RDS or J-RDS tests yielded the best threshold, (total) stereoblindness was defined as any true stereoacuity >=2000″ (which is simply the worst stereoacuity that we could measure). When the single-stimulus method yielded the best threshold, stereoblindness was defined as any true stereoacuity greater or equal to the 95^th^ percentile of our available stereoacuity data with that test. This procedure naturally results in a 5%-stereoblindness rate, the prevalence estimated by a couple of previous studies^9,10^.

Then, we investigated the best Randot criterion to classify participants between *stereoblind* and *not stereoblind* categories. For this purpose, we varied the stereoblindness criterion for the Randot-Circles test between 20″ and 2000″. Each criterion yielded a sensitivity and a specificity score. Resulting sensitivities and specificities are available online as an ROC figure (Supplementary Fig. S3). We define the best criterion as the one resulting in the largest positive predictive value (normalized to the 5%-prevalence) and the best negative predictive value in case of equality. The best criterion was any score >=400″ (level <=#1). That criterion for the Randot test (n = 61) gave a 73%-sensitivity (CI_0.95_ = [0.61–0.84]) and a 96%-specificity (CI_0.95_ = [0.90–1]; contingency table in Supplementary Table S4; summary in Table 2). Selecting totally-stereoblind participants requires a test with a high positive predictive value. The positive predictive value normalized for a 5%-prevalence is only NPPV = 49% (CI_0.95_ = [0.35–0.62]). Therefore, the positive predictive value of Randot Circles is too low for the low stereoblindness prevalence (error rate = 1 − NPPV = 51%). Even with a 100%-sensitivity, the test would suffer from its lack of specificity in detecting stereoblindness (error rate = 43%). The test sensitivity is also too low for screening stereoblindness: under normal viewing conditions, 27% of the stereoblind participants (1-sensitivity) can deceive the test.

**Table 2:** Test statistics for detecting stereoblindness (SB) and stereo-impairment (SI).

Randot	Circles – SB (≥400″)	Circles - SI (≥100″)	Shapes SB (nil)	Shapes SI (nil)
Sensitivity	0.73	0.46	0.73	0.56
Specificity	0.96	1	0.88	1
Normalized Positive Predictive Value	0.49	1	0.24	1
Normalized Negative Predictive Value	0.98	0.85	0.98	0.87

The table reports the test statistics of the Randot Circles and Randot Shapes. The header of the column also shows the best criterion for detection with the Randot.

#### Can we use the Randot-Circles test to diagnose stereo-impairment

The typical range of stereoacuity for neurotypical participants with a psychophysical test comparable to the Eyetracked-RDS and J-RDS tests that we used^46^ was 8″ to 106″ (37″ +/− 56″ with STD: 27″). Therefore, any person with a true stereoacuity higher (worse) than the upper limit (106″) can be considered *stereo-impaired* (this includes stereoblindness) when their best threshold is obtained with Eyetracked-RDS and J-RDS tests. When the single-stimulus method was used, stereo-impairment was defined as any true stereoacuity greater or equal to the 75^th^ percentile of our available stereoacuity data with that test. This procedure naturally results in a 25%-stereo-impairment rate, close to the prevalence estimated by Richards^47^.

To assess whether the Randot-Circles test is useful for diagnosing stereo-impairment, we varied the criterion for stereo-impairment from the Randot-Circles thresholds. Each criterion yielded a sensitivity and a specificity score (Supplementary Fig. S3). The best criterion (determined as above) was to attribute stereo-impairment for any score >= 100″ (level <= #4, n = 61). For that criterion, the test gave a 46%-sensitivity (CI_0.95_ = [0.32–0.59]) and a 100%-specificity (CI_0.95_ = [0.99–1]; contingency table in Supplementary Table S5; summary in Table 2). Detecting stereo-impaired participants requires a test with a high positive predictive value. The positive predictive value normalized for a 25%-prevalence of stereo-impairment was NPPV = 100% (CI_0.95_ = [0.99–1]) and the negative predictive value was 85% (CI_0.95_ = [0.75–0.95]). Therefore, the test is appropriate to select participants who are truly stereo-impaired (error rate = 1-NNPV = 0%), but not appropriate for screening purposes because of a lack of sensitivity in detecting stereo-impairment: under normal viewing conditions, 54% of stereo-impaired participants (1-sensitivity) can deceive the test.

#### Can we use the Randot-Shapes test to diagnose total stereoblindness

To determine true stereoacuity and stereoblindness, we used the same procedure as the one to detect stereoblindness psychophysically, described in the Randot-Circles section above. Then, we investigated the best possible criterion to classify participants between *stereoblind* and *not stereoblind* categories. For this purpose, we varied the stereoblindness criterion for the Randot-Shapes test between 20″ and 2000″. Each criterion yielded a sensitivity and a specificity score. Resulting sensitivities and specificities are available in an ROC figure (Supplementary Fig. S3). We defined the best criterion as the one resulting in the largest positive predictive value (normalized to the 5%-prevalence) and the best negative predictive value in case of equality. The best criterion was any zero (nil) score. That criterion (n = 52) gave a 73%-sensitivity (CI_0.95_ = [0.6–0.86]) and an 88%-specificity (CI_0.95_ = [0.78–98]; contingency table in Supplementary Table S6; summary in Table 2). The positive predictive value normalized for a 5%-prevalence was only NPPV = 24% (CI_0.95_ = [0.35–0.62]). Therefore, the positive predictive value of the Randot Shapes is too low for the low stereoblindness prevalence (error rate = 1-NPPV = 76%). Even with a 100%-sensitivity, the test would suffer from its lack of specificity in detecting stereoblindness (error rate = 70%). The test sensitivity is also too low for screening stereoblindness: under normal viewing conditions, 27% of the stereoblind participants (1-sensitivity) can deceive the test.

#### Can we use the Randot-Shapes test to diagnose stereo-impairment

To determine true stereoacuity and stereo-impairment, we used the same procedure as the one to detect stereo-impairment psychophysically, described in the Randot-Circles section above. To assess whether the Randot-Shapes test is useful for diagnosing stereo-impairment, we varied the criterion for stereo-impairment from the Randot-Shapes thresholds (the different numbers on Supplementary Fig. S3). Each criterion yielded a sensitivity and a specificity score for the test. The best criterion (determined as above) was any zero (nil) score (n = 52). For that criterion, the test gave a 56%-sensitivity (CI_0.95_ = [0.42–0.71]) and a 100%-specificity (CI_0.95_ = [0.99–1]; contingency table in Supplementary Table S7; summary in Table 2). The positive predictive value normalized for a 25%-prevalence of stereo-impairment was NPPV = 100% (CI_0.95_ = [0.99–1]) and the negative predictive value was 87% (CI_0.95_ = [0.77–0.97]). Therefore, the test is appropriate to select participants who are truly stereo-impaired (error rate = 1-NNPV = 0%), but not appropriate for screening purposes because of a lack of sensitivity in detecting stereo-impairment: under normal viewing conditions, 44% of the stereo-impaired participants (1-sensitivity) can deceive the test.

### Non-stereoscopic cues in the Butterfly Shape

#### Rotated-viewing (binocular) scores

The random-dot butterfly shape could be correctly identified by 39 of our 57 participants (68%; CI_0.95_ = [0.56–0.80]) in rotated viewing, showing that non-stereoscopic cues can be used to identify the shape. Depth (or more probably rivaldepth) was also reported 77% of the time (by 44 observers). Finally, both participants who were stereoblind according to psychophysical tests (n = 2, 100%) could pass this part of the test in rotated viewing, confirming the use of binocular non-stereoscopic cues.

The effect of presentation order played against our conclusion, since all participants but 3 started with rotated viewing but none of the 3 participants who started with the right-side-up viewing could identify the shape in rotated viewing. The participants with a score better than the median in rotated viewing were more likely to pass in normal viewing than the participants with a score worse than the median but the difference was not significant (80.8% vs. 66.7%; exact Fisher’s test p = 0.45).

### Non-stereoscopic cues in the Butterfly Circles

#### Patched-viewing (monocular) scores

**Figure 3:**
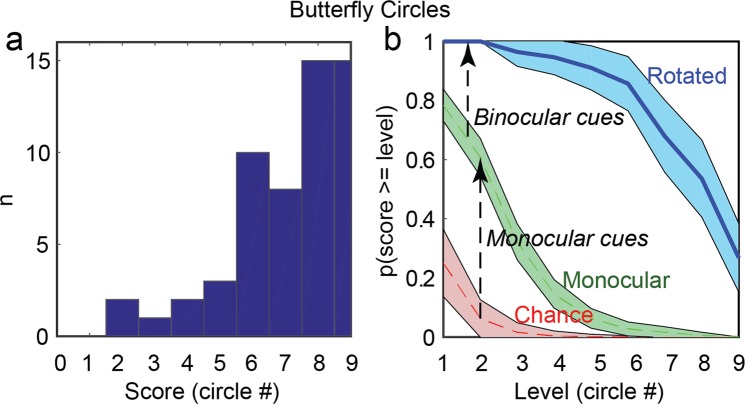
(**a**) Distribution of scores for the Butterfly-Circles test, in rotated viewing, for observers with typical vision. (**b**) Probability to pass at least the level in abscissa in rotated viewing (blue solid line). The probability to pass the level in abscissa by chance only is indicated with the red dashed line. The probability to pass the level monocularly is re-plotted from the literature with the green dashed line. Shaded areas depict the 95% confidence interval. Scores/levels for each reached circle: 1: 800″, 2: 400″, 3: 200″, 4: 140″, 5: 100″, 6: 80″, 7: 60″, 8: 50″, 9: 40″.

The circles portion of the Butterfly is identical to the Titmus Circles. We selected 6 studies^18–22,44^ that measured the Titmus Circles performance monocularly or with the same image to both eyes, with adults or children and with normal or pathological conditions (see Supplementary Table S8 for details and results). We pooled together all participants and expressed the proportions of success for each circle level (Fig. 3b). As is well known, monocular cues are clearly present in that portion of the test^18–22,44^.

#### Rotated-viewing (binocular) scores

Figure 3a shows the distribution of scores when the test is rotated by 90 degrees. Each score proportion (Fig. 3b) differed significantly from what is expected by chance (for all binomial tests, p = 0; n = 56; 1-β = 1) demonstrating the influence of non-stereoscopic cues at all disparity levels. Every participant could respond correctly at least up to the 400″-disparity level (circle #2) in rotated viewing. Fifteen out of 56 participants (27%, CI_0.95_ = [0.15–0.38]) could even correctly identify the item in depth at the smallest disparity (40″ – circle #9). Each score proportion also differed significantly from what is expected if only monocular cues were present in the test (Fig. 2b; n = 27; for all binomial tests, p < 10^−6^; 1-β = 1) demonstrating that binocular non-stereoscopic cues can provide additional information to deceive that portion of the test. The order of presentation, which was counterbalanced between participants, did not affect the performance in rotated viewing (Kruskal-Wallis Chi square = 0.95; p = 0.58; n = 56).

Finally, both participants who were stereoblind according to psychophysical tests (n = 2, 100%) could pass this part of the test in rotated viewing, confirming the use binocular non-stereoscopic cues.

## Discussion

It has long been known that stereoblind observers can deceptively pass stereotests relying on *monocular* cues present in some clinical stereotests^18–24,26,44^. The Randot test was designed to be immune to those cues and our measures confirm this fact.

Here, we hypothesized that both neurotypical and amblyopic observers might also be sensitive to *binocular non-stereoscopic* cues in stereotests. We provide evidence for the presence of binocular non-stereoscopic cues in the Randot and Butterfly stereotests by testing performance under rotated viewing and comparing it to performance under monocular viewing. The rotated viewing renders the binocular disparities vertical, and vertical disparities in this specific configuration do not produce stereoscopic depth. Large proportions of participants could identify the shapes in the random dots and find the circle in depth when the Randot and Butterfly tests were rotated 90 degrees, far above the chance level. Given participants could not pass the Randot test in the monocular viewing condition (confirming the lack of monocular cues), it demonstrates conclusively that the non-stereoscopic cues in the Randot are exclusively binocular non-stereoscopic cues. Rotated viewing of the Butterfly Circles yielded greater proportions of success at each level than what is largely reported in the literature for monocular viewing. This demonstrates that binocular non-stereoscopic cues provide additional information to deceive the Butterfly Circles. While non-stereoscopic cues are massively present to deceive the Butterfly Shape, we cannot conclude that binocular non-stereoscopic cues are present in that portion because of the absence of monocular viewing data.

Two recent studies^42,48^ used a rotated viewing condition to improve testing with the Titmus Fly and 19% to 47% of their sample passed the test in that condition. While this adds evidence for the presence of non-stereoscopic cues, it does not convincingly demonstrate the existence of binocular non-stereoscopic cues in the Titmus Fly. This is because the participants could have used monocular non-stereoscopic cues as no monocular viewing condition was used for comparison.

Interestingly, we show that amblyopic observers are less successful in using non-stereoscopic cues in these tests than neurotypical observers. It is possible that amblyopic suppression prevents our amblyopic observers from experiencing binocular rivalry, diplopia/confusion, luster or rivaldepth, but this requires further investigation.

We do not mean to imply that the Randot test is invalid. The Randot Circles test measures a perceptual ability that is highly correlated with the stereoscopic ability measured in our psychophysical tests. Despite this, we show that the Circles and Shapes portions of the Randot test cannot be used to detect total stereoblindness with a high normalized predictive value because of their lack of specificity. The test is also inappropriate for screening for stereoblindness because of its low sensitivity. It can however be used to detect stereo-impairment (level <= #4, stereoacuity >= 100″ for the Circles and nil for the Shapes) with a perfect normalized predictive value. However, its low sensitivity (46% for the Circles and 56% for the Shapes) makes it inappropriate for screening stereo-impairment. We found only two stereoblind participants in the sample that we tested with the Butterfly test and therefore we could not reliably assess its ability to diagnose stereoblindness. The Butterfly Circles yielded such small variations (scores ranged from 40″ to 50″) that we could not study its correlation with psychophysical tests. Finally, we manipulated interocular contrast difference and relative interocular location of the stimuli to ensure the optimal conditions for fusion in our amblyopic patients when using psychophysical tests. Only patients capable of fusion under those conditions were recruited. Therefore, we can only generalize our results to participants with possible fusion under the conditions that we used.

In theory, our psychophysical tests were devoid of useful non-stereoscopic cues. To ensure the absence of monocular cues, we carefully followed standards in the field, by using brief presentations, dynamic random-dot stereograms^49^, or by adding a horizontal random jitter to each of the stimuli to compare when edges were present^50^. Even if binocular non-stereoscopic cues were present in our psychophysical tests, they were unlikely to be useful because the tasks relied on ordering items in depth rather than detecting an odd item or a specific shape. Binocular non-stereoscopic cues do not provide the stereoscopic information required for a depth-ordering task. Special efforts were made to avoid delta-vergence in the Eyetracked-RDS when testing long stimulus exposures.

In theory, binocular non-stereoscopic cues are created any time disparities are present. These cues could be used to pass a stereotest that requires an individual to detect a difference between depth objects (e.g. identifying a concealed shape in a random-dot stereogram or an odd item) rather than to order them in depth. Most clinical stereotests fall under that umbrella.

To prevent the use of binocular non-stereoscopic cues, we recommend tasks in which the participant is asked to order objects in depth, e.g. to tell whether one object (e.g. a textured shape) is in front of another object (e.g. a textured background) or whether it is behind. Dynamic random-dot stereograms are the safest way to avoid monocular cues.

It is possible that the poor efficacy of the clinical stereotests for screening children with binocular anomalies^14^ is related to the presence of binocular non-stereoscopic cues. In the future, we hope that researchers will examine other stereotests for binocular non-stereoscopic cues or design stereotests that are free of binocular non-stereoscopic cues and determine their screening efficiency.

## Electronic supplementary material

Supplementary information

## Data Availability

Data are available on Mendeley Data at 10.17632/g6z5svv6jx.2 ^51^.
