# Supplementary material for: Binocular non-stereoscopic cues can deceive clinical tests of stereopsis
Source: Sci Rep. 2019 Apr 8;9:5789. doi: 10.1038/s41598-019-42149-2 (PMC6453951; doi:10.1038/s41598-019-42149-2)
Supplement: Supplementary file 1 — Supplementary information [file 41598_2019_42149_MOESM1_ESM.pdf]

TITLE

Binocular non-stereoscopic cues can deceive clinical tests of stereopsis.

AUTHOR LIST & AFFILIATIONS

Adrien Chopin[1,2,*], Samantha Wenyan Chan[2], Bahia Guellai[3], Daphné Bavelier[1], Dennis Michael Levi[2]

1 Department of Psychology and Education Sciences, University of Geneva, Switzerland

2 School of Optometry, University of California, Berkeley, USA

3 Laboratoire Ethologie, Cognition, Développement, Université Paris Nanterre, France

SUPPLEMENTARY INFORMATION

Supplementary Methods

Participants

All observers in samples 1 to 3 (including one author) were included on the basis of typical vision and we excluded no participants. Samples 1 and 2 include participants whose data was partially previously published for a different analysis[1,2]. Sample 2 includes 13 professional dressmakers and is described in detail elsewhere[1,2]. Sample 3 includes neurotypical observers (with no exclusion) recruited with an ad.

Samples 4 and 5 are composed of 19 amblyopic observers recruited from a list of previous patients with binocular pathologies in an eye clinic (initial n = 955). After a screening by an optometrist, they were called for inclusion if a diagnosis of strabismic, anisometropic or mixed amblyopia could be made, and no exclusion criteria applied to them (n=412, see recruitment diagram in Supplementary Fig. S1). A diagnosis of amblyopia could be made if the two following criteria applied: (1) at least a 2-line interocular acuity difference with best-corrected vision after excluding any eye disease, (2) presence of a clear amblyogenic cause (strabismic if any tropia, anisometropic if any refraction difference >1 spherical-equivalent diopter, or mixed if the two criteria applied). Other exclusion criteria included a tropia >30Δ, any monocular visual acuity worse than 20/200, age >65 years, binocular amblyopia, nystagmus, intermittent exotropia, diplopia, a history of traumatic brain injury or severe neurological or psychiatric disorder, current psychoactive medication, unreachable or language barrier, any MRI contra-indication (we intend to scan their brain), if they were not interested or already recruited in another training experiment. Only 30 were eligible and interested in participating. Seven chose another experiment in the lab. After testing the participants with the Diplopia-Suppression test, four were excluded because we could not obtain fusion in the best conditions (see procedure and recruitment diagram of Supplementary Figure 1). An additional participant with amblyopia was discovered later and added to Sample 6. Clinical details of the amblyopic participants are provided in Supplementary Table 3.

All participants in Sample 6 had typical vision (including one author) except for one additional amblyopic patient. They were all recruited among students with no specific inclusion criteria. Samples 1-3 were recruited with an ad. Samples 3 and 6 followed convenience sampling while samples 1, 2, 4 and 5 followed consecutive sampling.

Stimuli and Material

All programs used Matlab and PsychToolbox with a Macintosh Power PC computer. All psychophysical tests were designed to be free of non-stereoscopic cues and we used a threshold of 2000” (33.33 arcmin) to denote stereoblindness (except for the single-stimulus-method test, which allows larger disparities). Participants were tested on clinical stereotests before being tested on psychophysical stereotests (at the same session), so that the assessors had no access to the true stereo-acuity before performing the clinical test.

Stimuli for the clinical stereotests

The Randot stereotest consists of three sections: circles, shapes and animals. The Circles are a series of graded circles of different level of disparity, grouped by 3, and the groups are ordered by descending disparities. The background of each group is made of random dots. The Shapes consists in groups of four random-dot squares containing stereograms. Three out of four depict a simple hidden shape in depth. The animal section was not used.

The random dot Butterfly stereotest also consists of three sections: circles, a shape and animals. The Circles are similar to the Randot Circles except that they are grouped by four. The shape is a large unique square of random dots in which a hidden butterfly shape stereogram is hidden. The animal section was not used.

Stimuli for the Diplopia-Suppression Test

In the Diplopia-Suppression Test, frames were 9-degree large and the central circle has a 3-deg diameter. The background had a luminance of 40 cd/m^2. Fixation dot was 0.2-deg diameter and small circles were 0.6-deg large, while dots were 0.25-deg large.

Stimuli for the J-RDS psychophysical stereotest

Dynamic random-dot stereograms were presented on a black background (around 0.5 cd/m^2). White was defined as 30 cd/m^2. Random-dots stereograms were made of white dots (0.28-deg size, 15%-density) whose location was randomly chosen. The white fixation point (0.2-deg diameter) was in the center of a white fusion frame (13.6-deg large). The stereogram depicted a target square (1.6-deg large) in the middle of a larger background square (3.2-deg large). The disparity of the target square was varied on each trial (among the values 0”, 102”, 204”, 408”, 816”, either crossed or uncrossed). The background square disparity stayed fixed (0”-fixation disparity). The fixation dot and fusion frame were always in the depth plane of the background square. Every 100 ms, a new set of random dots depicting the same disparity configuration was displayed (dynamic).

The stimuli for the J-RDS were presented on a Sony Triniton Multiscan G500 monitor (1280x1024pp; 35x29.5cm; refresh rate 60 Hz) and viewed through a 4-mirror stereoscope, at a distance of 68 cm.

Stimuli for the Eyetracked-RDS psychophysical stereotest

The stimuli of the Eyetracked-RDS were similar to the ones in the J-RDS with the following changes. The background was 15 cd/m^2, white was defined as 30 cd/m^2 and black around 0.5 cd/m^2. A black circle (2.5-deg diameter) surrounded a black fixation dot (0.25-deg diameter). Half of the dots of the random-dot stereograms were white and the other half were black. However, the non-black dots of the target square were blue. The fusion frame was black. Only the background-square random-dots had a disparity varying from trial to trial, all other displayed elements staying at a 0”-fixation disparity. Dot density was 20% and size was 0.25 degree of visual angle. Every 250 ms, a new set of random dots depicting the same disparity configuration was displayed (dynamic).

Stimuli for the Eyetracked-RDS were presented on a NEC SuperBright Diamondtron MultiSync FP2141 (1600x1200pp; 39x29.5cm; refresh rate 60 Hz) at a viewing distance of 75 cm. Eyetracking was achieved with an Eyelink-II eyetracker running on a separate computer with the ViewPoint software.

Stimuli for the single-stimulus method psychophysical stereotest

The stimuli and setup for the single-stimulus method are described in detail elsewhere[2]. In summary, two white lines at different depth were flashed in front a white fixation dot, on a black background.

Procedure for the clinical stereotests

We followed manufacturer’s instructions for the Randot and Butterfly tests with a few exceptions. Randot Circles and Shapes were scored independently. For the Randot Shapes, the manufacturer instructions are unclear on the number of shapes necessary to score one level of disparity. We decided to validate the level if three shapes (or absence of shape) out of four could be recognized. We tested each level independently and noted the best score. Butterfly Circles and Shape were scored independently. For the Butterfly Shape, all participants who could recognize the hidden shape could also point to the wings and abdomen of the butterfly. Therefore, we separated them between pass and fail. Participants were invited to take their time and encouraged to try to respond. They were discouraged to tilt their head and the experiment constantly verified that they were not tilting their head. The order of presentation is available in the Supplementary Table 2.

Procedure for the Diplopia-Suppression Test

All participants who were tested on the J-RDS or the Eyetracked-RDS stereotests had to first pass the Diplopia-Suppression Test. The test started with calibrating the stereoscope mirror position by equaling the accommodation and vergence distances. Calibration was achieved by presenting lines viewed through the mirrors and having participants align them with sticks viewed above the mirrors. After calibration, participants were presented with a black-and-white frame in the middle of which was shown a monocular circle and a binocular fixation dot. Half of the monocular circle was presented to one eye and the other half to the other eye. Observers were asked to adjust the vertical relative inter-ocular location of the stimulus presented to each eye so that the binocular frame appears fused and the two monocular half circles appear vertically aligned. The inter-ocular contrast, and if necessary the horizontal location, could also

be varied (with one eye's contrast fixed at 96%) as long as the binocular frame was not fused or the two half circles suppressing each other. After that, a binocular circle replaced the half circles and 6 smaller circles appeared, three on each side of the circle. In some of those small circles, one binocular dot was presented (min: 0 and max: 2). Participants were asked to report the number of dots perceived in each of 10 trials, numbers that we used to compute the proportion of trials in diplopia, i.e. in which the number of perceived circles is twice the number of actual circles. Finally, participants were asked to detect whether one dot was present or not in all six circles. The dot could be monocular in the left eye (15 trials) or monocular in the right eye (15 trials). We used the responses to calculate a detection sensitivity d' for each eye and consider it a measure of each eye inter-ocular suppression. If less than 20% of the trials were diplopic and all d' values were above 1, the Diplopia-Suppression test was deemed passed and the inter-ocular stimulus locations and contrasts were saved. For each trial of the test, a sound was produced after each key-press. Inter-stimulus interval was 200ms. If fusion could not be achieved, the test was attempted at a 150-cm distance.

Procedure for the J-RDS psychophysical stereotest

Some participants were tested on the psychophysical stereotest J-RDS (one participant of sample 1, 3 participants in sample 3, all participants in sample 4). After passing the Diplopia-Suppression Test, participants started the J-RDS. Optimal relative ocular location and contrast for fusion were retrieved from the Diplopia-Suppression Test. The location was used for the J-RDS stimuli and the contrast for the fusion frames and fixation dot. The task consisted in judging whether the target square was in front or behind the background square and to report each trial's judgment using the keyboards keys. A sound was produced after each key-press. Disparities of the target square were chosen using the method of constant stimuli while the background square disparity was fixed. 180 trials were run to calculate each 75%-correct threshold. Each trial started with a fixation dot and fusion frames and the participant initiated stimulus presentation with a key. After the stimulus presentation, a black screen was shown until the participant responded. Thresholds were measured two times, once with a stimulus-presentation duration of 200 ms and once with a duration of 2000 ms.

Procedure for the Eyetracked-RDS psychophysical stereotest

Some participants were tested on the psychophysical eyetracked-RDS test (all participants in Sample 5, one in Sample 1, 5 in Sample 3). After passing the Diplopia-Suppression Test, participants started the Eyetracked-RDS test. The test is an eyetracked version of the J-RDS test with an emphasis on practice and a target random-dot square in the same depth plane as the fixation dot and fusion frame. Thus, the procedure was similar to the J-RDS test with the exception of the following changes. Before the actual test, several practices were run. All of the practices had relevant auditory feedback (high-pitch beep for correct responses, low-pitch beep otherwise) and each of them could be repeated if the participant had not reach 61%-correct. During the first practice, 2 repeats of each disparity were measured with large disparities (137.5", 275", 412.5", 550", and 1100", crossed and uncrossed) for a stimulus duration of 2000 ms. During the second practice, 2 repeats of each large disparity were measured for a stimulus duration of 1000 ms. During the third practice, 2 repeats were measured with the test disparities (0", 34.5", 69", 137.5", 275", and 550", crossed and uncrossed) for a stimulus duration of 1000 ms. For the fourth practice, 2 repeats of each test

disparity were measured for a stimulus duration of 200 ms. Then, the 200ms-test was run for the test disparities with a 200-ms stimulus-duration (16 repeats of each disparity), using an irrelevant auditory feedback (constant beep). On a second session, participants ran the Diplopia-Suppression Test again, followed by a practice in which participants were trained to fixate inside the fixation circle only during 2000-ms trials. Visual feedback of their eyetracked fixation was produced on the screen, with a dot whose color was green when close to the fixation dot and red when close to the fixation circle limit. If participants fixated outside of the circle, the trial was interrupted, a loud noise was produced and the trial was repeated. A second practice was given in which participants were instructed to manage both the fixation task with visual feedback and the depth task (2 repeats of each test disparities) for 2000-ms trials. Finally, the task was administered for 16 repeats of each test disparity for 2000-ms trials, with no visual feedback and irrelevant auditory feedback.

Psychophysical stereotest: single-stimulus method

Some participants were tested on the single-stimulus-method stereotest (all participants in Sample 2 and one in Sample 1). The procedure to run this stereotest was described elsewhere in detail[2]. In summary, the vergence bias and vergence noise of the participants was first tested using a Nonius-line method, after which their stereoacuity for absolute and relative disparities was separately assessed. The single-stimulus method involves comparing the distance of the current stimulus with the average of the distances for the past stimuli.

Analysis

We were always able to yield a score on each test. Participants who were measured at least with either the Randot or the Butterfly stereotest rotated and right-side up/upside-down were included (no exclusion occurred at that stage). Exact sample size was determined by the protocols of other experiments from which samples were pooled together: we merged all available data to answer the experimental question.

Supplementary References

1. Chopin, A., Levi, M. D. & Bavelier, D. Dressmakers show enhanced stereoscopic vision. *Sci. Rep.* **7,** (2017).
2. Chopin, A., Levi, D. M., Knill, D. & Bavelier, D. The absolute disparity anomaly and the mechanism of relative disparities. *J. Vis.* **16,** 1–17 (2016).

Supplementary Figures

Supplementary Figure S1: diagram of the recruitment procedure

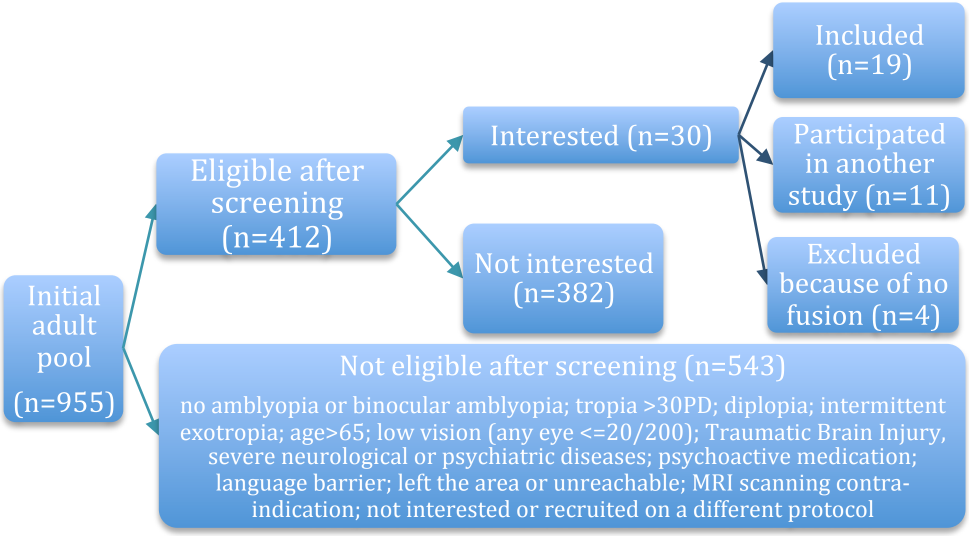

Supplementary Figure S2: best psychophysical stereoacuity vs. Randot Circles stereoacuity

Each circle represents one to eight participants, depending on the size of the circle. The black dashed line indicates the same stereoacuity for the psychophysical test and the clinical test, in arcsec (the smaller the value is, the better the stereoacuity, the more precise at discriminating stereoscopic depths). The red continuous line is the robust linear regression line (bisquare weighting) of the best psychophysical stereoacuity over the Randot Circles stereoacuity. The orange line is the equality line.

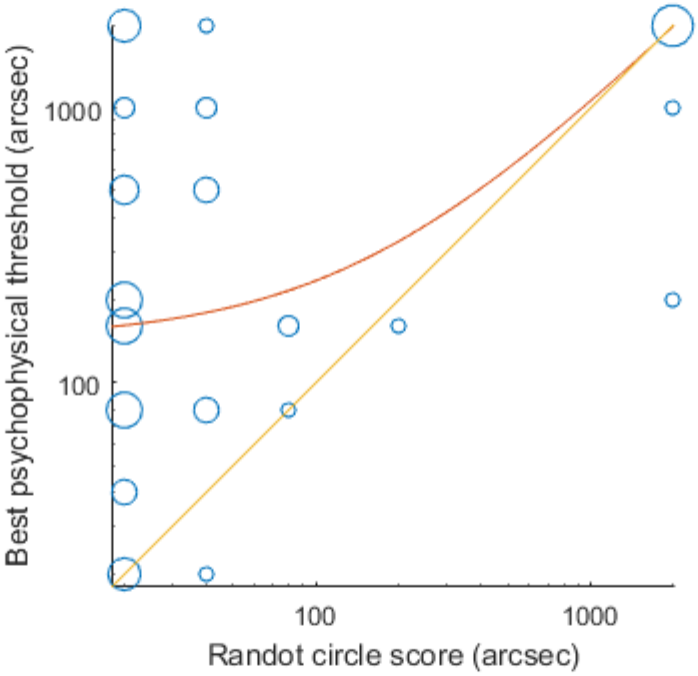

Supplementary Figure S3

ROC with various criteria for detection stereoblindness (red lines) and stereo-impairment criterion (blue lines) from the score on the Randot-Circles test (dashed lines) or the Randot-Shapes test (solid lines). Equal sensitivity and [1-specificity] are on the black line. There is one number and circle for each particular criterion on the Randot test. A score worse or equal to it would be deemed stereoblind (red) or stereo-impaired (blue).

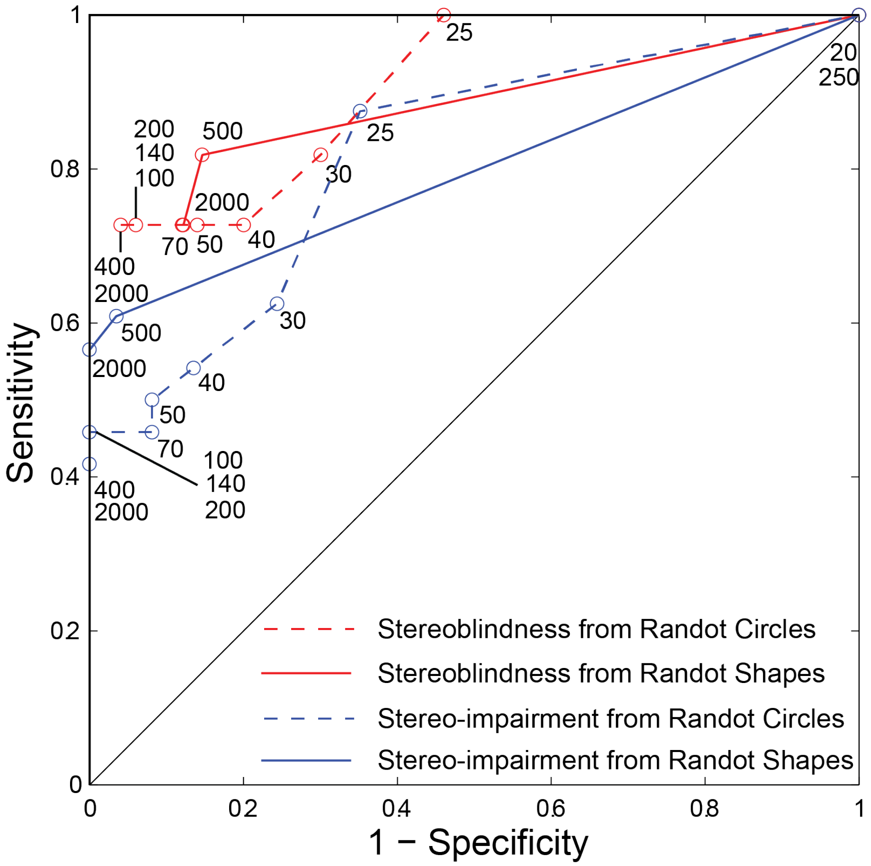

SUPPLEMENTARY TABLES

Supplementary Table S1: Description of the samples

For each sample, the table provides the city of recruitment, the year of recruitment, the number of neurotypical participants and amblyopic participants, the age range and age mean, and the proportion of female observers in the sample.

	Location	Year	Nb of neurotypical / amblyopic	Age range (and mean)	% Female
Sample 1	Geneva	2012	23/0	20-37 (29)	43
Sample 2	Geneva	2013	34/0	19-35 (25)	41
Sample 3	Berkeley	2014-2015	7/0	19-23 (21)	43
Sample 4	Berkeley	2014-2015	0/2	20-21 (20.5)	50
Sample 5	Berkeley	2014-2017	0/17	20-61 (34)	82
Sample 6	Berkeley	2017-2018	26/1	18-65 (23.8)	63

Supplementary Table S2: tests, conditions and results by participant

Participant: * indicates author. Amblyopia status: a=amblyopic, n=neurotypical; Butterfly shape: p=pass all the pointings (stereoacuity: 700"), f=fail the shape recognition (no stereoacuity); Butterfly and Randot circles order: the Butterfly and Randot shapes were tested rotated before right-side up except for the Butterfly of participants 1, 3 and 9; when order is coded: 1=rotated, right-side up, upside-down; 2=rotated, upside-down, right-side up; 3=right-side up, rotated, upside-down; 4=right-side up, upside-down, rotated; 5=upside-down, right-side up, rotated; 6= upside-down, rotated, right-side up; a=rotated, right-side up, upside-down; b=rotated, upside-down, right-side up; c=right-side up, rotated, monocular; d=right-side up, monocular, rotated; e=monocular, right-side up, rotated; f= monocular, rotated, right-side up; all other number indicate scores in arcsec, no=stereoacuity not measureable. Green cells indicates thresholds below 140”, orange indicates thresholds equal or greater than 140” and below 2000”, and red not measurable thresholds or threshold above 2000”. Color indicates normal stereopsis (green), stereo-impairment (orange) or stereoblindness (red). It depends on best criterion for stereoimpairment and stereoblindness defined in the text (Randot Circles >=100" and >=400" respectively; Randot Shapes not measurable; J-RDS/Eyetracked-RDS >=106" and >=2000"; Single Stimulus method >=832" and >=4489").

Participant	Sample	Amblyopia status	Butterfly shapes: rotated	Butterfly shapes: right-side up	Butterfly circles: order	Butterfly circles: rotated	Butterfly circles: right-side up	Butterfly circles: upside-down	Randot shapes: rotated	Randot shapes: right-side up	Randot shapes: upside-down	Randot shapes: monocular	Randot circles: order	Randot circles: rotated	Randot circles: right-side up	Randot circles: upside-down	Randot circles: monocular	Psychophysics: Best J-RDS	Psychophysics: Best Eyetracked-RDS	Psychophysics: Single Stimulus Method
1	1	n	f	p	4	80	40	40												
2*	1	n	p	p	4	100	40	40	250	250	250	2000	1	100	20	70	2000	20	38	85
3	1	n	f	p	4	50	40	40												
4	1	n	f	p	4	50	40	40												
5	1	n	f	p	4	50	40	40	2000	250			3	100	40	20				
6	1	n	f	p	4	40	40	40												
7	1	n	f	p	4	400	40	40												
8	1	n	p	p																
9	1	n	f	p	3	40	40	40												
10	1	n	f	p	3	50	40	40	250		250		4	50	20	40				
11	1	n	p	p	1	100	40	40	250		250		2	70	30	20				
12	1	n	f	p	1	60	40	40												
13	1	n	p	p	2	60	40	40												
14	1	n	f	p	3	200	40	50												
15	1	n	p	p	5	40	40	50												
16	1	n	p	p	6	40	40	40												
17	1	n	f	p	1	80	60	60												
18	1	n	p	p	2	50	40	50												
19	1	n	p	p	3	140	40	40												
20	1	n	p	p	4	140	40	40												
21	1	n	p	p	5	80	40	40	250		250		2	40	20	70				
22	1	n	f	p	5	50	40	40	2000		250		2	140	25	20				
23	1	n	p	p	5	40	40	40												
24	2	n	p	p	1	60	40	40	250	250			4	70	20	30				93
25	2	n	p	p	6	40	40	40	250	250			2	100	30	70				4932
26	2	n	p	p	1	40	50	60	250		250		3	70	25	140				3040
27	2	n	p	p	2	40	40	40	2000		250		6	40	25	30				3162
28	2	n	p	p	1	60	40	40	250	250			5	50	20	70				109
29	2	n	p	p	3	80	40	40	250		250		6	70	20	30				131
30	2	n	f	p	5	40	40	50	2000	250			1	200	30	100				629
31	2	n	f	p	6	40	40	50	500		250		2	20	25	30				65
32	2	n	p	p	1	50	40	40	500		250		3	70	20	70				292
33	2	n	p	p	2	400	60	50	250	250			4	200	30	70				1194
34	2	n	f	p	3	50	50	40	250		250		5	70	70	70				179
35	2	n	p	p	5	50	40	40	250	250			1	25	20	20				241
36	2	n	p	p	6	60	50	50	500	250			2	70	40	30				630
37	2	n	p	p	2	50	40	40	250		250		4	70	25	40				108

38	2	n	p	p	4	50	40	40	250	250			5	40	20	20				147
39	2	n	f	p	1	100	40	40	2000		250		6	40	20	20				1715
40	2	n	p	p	2	50	40	40	250	250			1	70	20	20				625
41	2	n	p	p	3	80	40	40	500		250		2	100	20	70				1142
42	2	n	p	p	4	40	40	40	250	250			3	40	30	30				453
43	2	n	f	p	5	80	40	40	250		250		4	70	40	30				1033
44	2	n	p	p	6	60	40	40	2000	250			5	40	20	20				345
45	2	n	p	p	5	80	40	40	250		250		6	40	20	25				95
46	2	n	p	p	6	50	40	40	250	250			1	40	20	20				214
47	2	n	p	p	2	40	40	40	250	250			3	30	20	20				138
48	2	n	p	p	2	50	40	40	250	250			5	40	25	25				900
49	2	n	p	p	3	80	40	40	250		250		6	100	25	20				12060
50	2	n	p	p	4	60	40	40	500	250			1	70	70	25				165
51	2	n	f	p	6	80	40	40	500	250			3	50	20	20				179
52	2	n	p	p	1	40	40	40	250		250		4	20	20	30				344
53	2	n	p	p	2	60	40	40	250	250			5	40	20	40				221
54	2	n	p	p	3	80	40	40	500		250		6	140	25	20				447
55	2	n	p	p	4	40	40	40	250	250			1	40	20	40				174
56	2	n	p	p	6	40	40	40	250	250			3	70	20	20				395
57	2	n	p	p	1	50	40	40	250		250		4	30	20	40				123
58	3	n											3	70	25				57	
59	3	n											3	20	20				17	
60	3	n											3	200	20				49	
61	3	n											3	50	20				431	
62	3	n											3	50	30			71	61	
63	3	n											3	30	20			20		
64	3	n											3	25	20			27		
65	4	a											3	25	20			33		
66	4	a											3	100	30			20		
C#1	5	a							2000	500			3	400	40				69	
C#2	5	a							2000	2000			3	200	200				156	
C#3	5	a							2000	2000			3	2000	2000				214	
C#4	5	a							2000	2000			3	100	50				114	
C#5	5	a							500	250			3	100	20				21	
C#6	5	a							2000	250			3	200	70				100	
C#7	5	a							2000	2000			3	20	25				109	
S#1	5	a							2000	2000			3	2000	2000				2000	
S#2	5	a							2000	2000			3	2000	2000				2000	
S#3	5	a							2000	2000			3	2000	2000				2000	
S#4	5	a							2000	2000			3	2000	2000				2000	
S#5	5	a							2000	2000			3	2000	2000				2000	
S#6	5	a							2000	2000			3	2000	2000				1414	
T#1	5	a							2000	2000			3	50	25				2000	
T#2	5	a							2000	500			3	2000	2000				2000	
T#3	5	a							2000	2000			3	2000	2000				2000	
T#4	5	a							2000	2000			3	2000	2000				2000	
001*	6	n							500	250		2000	2	50	20		2000			
0010	6	n							250	250		2000	1	50	20		2000			
0014	6	n							250	250		2000	5	30	20		2000			

0011	6	n	500	250	2000	6	50	20	2000
0008	6	n	2000	250	2000	2	50	20	2000
0012	6	n	2000	250	2000	1	200	25	200
0015	6	n	250	250	2000	3	30	20	2000
0016	6	n	2000	250	2000	4	50	20	200
0017	6	n	2000	250	2000	5	100	20	2000
0019	6	n	500	250	2000	2	70	70	200
0020	6	n	500	250	2000	1	140	140	2000
0021	6	n	500	250	2000	3	50	20	400
0022	6	n	250	250	2000	4	70	20	2000
0023	6	a	250	250	2000	5	2000	20	140
0024	6	n	250	250	2000	2	70	20	2000
0025	6	n	250	250	2000	1	2000	20	2000
0026	6	n	2000	250	2000	3	70	20	2000
0027	6	n	250	250	2000	4	20	20	2000
0028	6	n	500	250	2000	5	140	40	2000
0029	6	n	2000	250	2000	6	70	50	2000
0030	6	n	500	250	2000	3	140	70	2000
0031	6	n	2000	250	2000	2	200	50	2000
0032	6	n	500	250	2000	1	70	50	2000
0033	6	n	500	250	2000	4	70	50	100
0035	6	n	250	250	2000	6	50	20	2000
0036	6	n	500	250	2000	2	50	20	2000
0037	6	n	250	250	2000	1	30	20	140

Supplementary Table S3: Clinical characteristics of the amblyopic patients

For each patient, the table provides Sample # / patient #, age/sex, amblyopia type and amblyopic eye, stereoacuity measured with the Randot, refraction; visual acuities, tropia and clinical comments. Patients were selected first through a Berkeley Eye Center and further screened for retinal or eye diseases, and tested for refraction (autorefractor followed by subjective refraction), adjusted with best subjective correction, and further tested with the Sloan or Bailey-Lovie charts, Cover Test and Randot test. Patients that we could not determine the amblyogenic factor, who took psychoactive medication, or had psychiatric or neurological disorder, or showed nystagmus, diplopia or history of it, intermittent tropia, tropia larger than 30Δ, or an eye with acuity worse than 20/200 were excluded. Any amblyopia with tropia was considered strabismic amblyopia, with anisometropia of at least 1DS was considered anisometropic amblyopia, and with both, was considered mixed amblyopia.

S#-P#	Age/ Sex	Amblyo-pia type / eye	Randot stereo-acuity	Refraction at 3m distance	Visual Acuity at 3m distance	Tropia	Comments
S4-1	22/M	Aniso / OD	25”	OD: +2.75−1.00×170<br>OS: +0.25 DS	OD: 20/50+1<br>OS: 20/12.5+2	Ortho	
S4-2	21/F	Mixed / OS	140”	OD: -1.25−0.75×168<br>OS: +0.75−3.75×164	OD: 20/16-2<br>OS: 20/32+2	Dist.: Ortho<br>Near: 8ΔLXT	
S5-1	61/F	Mixed / OS	40"	OD: -3.25 DS<br>OS: -4.00 DS	OD: 20/25-2<br>OS: 20/50-2	4ΔLHypoT	
S5-2	20/F	Aniso / OS	200"	OD: Plano<br>OS: 3.00−1.00×15	OD: 20/16+2<br>OS: 20/63-2	Ortho	
S5-3	61/F	Aniso / OS	None	OD: +2.00−0.25×134<br>OS: +6.25−0.50×74	OD: 20/12.5-2<br>OS: 20/63-2	Ortho	
S5-4	41/F	Mixed / OS	50"	OD: -5.50−0.25×26<br>OS: +1.00−3.50×158	OD: 20/20-1<br>OS: 20/40+2	Dist.: 6ΔLEP<br>Near: 6ΔLET	Slight nystagmus when looking down
S5-5	24/F	Aniso / OS	20"	OD: +7.25 DS<br>OS: +9.00−0.50×155	OD: 20/20-2<br>OS: 20/50+1	Ortho	Recovered stereo from previous training
S5-6	27/M	Aniso / OS	70"	OD: -1.00 DS<br>OS: -0.50−0.25×25	OD: 20/16-2<br>OS: 20/40+2	Ortho	Intermittent 8ΔLXT at near
S5-7	26/F	Mixed / OS	25"	OD: -14.00−1.00×20<br>OS: -12.00−2.00×175	OD: 20/20-1<br>OS: 20/40-1	8ΔLET	
S5-8	50/F	Aniso / OS	None	OD: +1.00 DS<br>OS: +2.25−1.50×120	OD: 20/25+1<br>OS: 20/100-2	Ortho	
S5-9	23/F	Mixed / OS	200"	OD: Plano−0.50×165<br>OS: 2.75−1.00×155	OD: 20/20-2<br>OS: 20/100+1	Dist.: 8ΔLXT<br>Near: 4ΔLXT	
S5-10	26/M	Strab / OD	None	OD: +1.75−1.75×180<br>OS: +1.25−2.00×180	OD: 20/160-1<br>OS: 20/20-1	8ΔRHyperT	
S5-11	22/F	Mixed / OS	None	OD: Plano<br>OS: +1.75−0.75×170	OD: 20/16-1<br>OS: 20/40	Dist.: 9ΔLET<br>Near:12ΔLET	
S5-12	32/F	Strab / OS	None	OD: -2.00−0.75×150<br>OS: -2.00−0.75×140	OD: 20/20<br>OS: 20/50	Dist.: 6ΔLET<br>Near: 4ΔLET	
S5-13	21/F	Mixed /OD	None	OD: +0.75−0.25×170<br>OD: -1.25 DS	OD: 20/40-1<br>OS: 20/16+1	Ortho	Microstrabismic (from 4ΔBO test)
S5-14	25/F	Aniso / OS	25"	OD: -0.25−0.75×180<br>OS: +4.50−5.25×180	OD: 20/20+2<br>OS: 20/63+2	Ortho	
S5-15	30/F	Strab / OS	500"	OD: +0.25−0.50×45<br>OS: -0.50−1.00×143	OD: 20/25-1<br>OS: 20/80	Dist.: Ortho<br>Near: 2ΔLET	Microstrabismic
S5-16	50/M	Aniso / OS	None	OD: Plano−0.50×95<br>OS: +4.00−1.50×80	OD: 20/20+2<br>OS: 20/40+2	Ortho	
S5-17	44/F	Mixed / OS	None	OD: Plano<br>OS: +2.25−2.00×70	OD: 20/12.5-1<br>OS: 20/50-1	Ortho	Microstrabismic (from 4ΔBO test)
S6-1	19/F	Aniso / OS	Titmus 9/9	OD: -1.75 DS<br>OS: +5.00-1.25×32	OD: 20/15<br>OS: 20/50	Ortho	

Supplementary Table S4: Contingency table for diagnosis of stereoblindness with the Randot-Circles

Randot stereotest was positive if the Circles test gave an acuity of 400 arcsec or worse.

	Positive stereoblindness	Negative stereoblindness
Positive Randot test	8	2
Negative Randot test	3	48

Supplementary Table S5: Contingency table for diagnosis of stereo-impairment with the Randot-Circles

Randot stereotest was positive if the Circles test gave an acuity of 100 arcsec or worse.

	Positive stereo-impairment	Negative stereo-impairment
Positive Randot test	11	0
Negative Randot test	13	37

Supplementary Table S6: Contingency table for diagnosis of stereoblindness with the Randot-Shapes

Randot stereotest was positive if the Shapes test gave an acuity of 2000 arcsec or worse.

	Positive stereoblindness	Negative stereoblindness
Positive Randot test	8	5
Negative Randot test	3	36

Supplementary Table S7: Contingency table for diagnosis of stereo-impairment with the Randot-Shapes

Randot stereotest was positive if the Shapes test gave an acuity of 2000 arcsec or worse.

	Positive stereo-impairment	Negative stereo-impairment
Positive Randot test	13	0
Negative Randot test	10	29

Supplementary Table S8: studies to determine the presence of monocular cues

Pop. Column: different populations (N = neurotypical, BIN = binocular dysfunctions, STR = strabismus, MS = monofixation syndrome), Age column: adults (A) or children (C), n: number of participants, View.: viewing condition (M = monocular, S1 = identical image to both eyes, S2 = after failing the Circles, patients were asked to use lateral offset under binocular viewing), * we removed group 3 who had very different instructions than the manufacturer's ones.

Studies	Pop.	Age	n	View.	Proportions of participants reaching the level (%)								
Titmus Circles					*800"*	*400"*	*200"*	*140"*	*100"*	*80"*	*60"*	*50"*	*40"*
Chance level					*25*	*6*	*1.6*	*0.4*	*0.1*	*0*	*0*	*0*	*0*
Hahn et al., 2010	N	A	100	M	60	35	5	3	0	0	0	0	0
Levy & Glick, 1974	N	A	10	M	100	100	100	?					
Cooper & Warshowsky, 1977 *	N	A	39	S1	90	85	61	46	28	10	8	3	0
Hall, 1982	N	A	12	M	60	60	60	18	0	0	0	0	0
Simons & Reinecke, 1974	BIN	A	11	S2	100	100	100	?					
Hahn et al., 2010	STR	A	33	M	100	62	11	7	2	2	0	0	0
Clarke & Noel, 1990	MS	C	22	M	100	100	54	23	4	?			
Total n passing					*158*	*120*	*65*	*31*	*15*	*5*	*4*	*1*	*0*
Total n					*227*	*227*	*227*	*206*	*206*	*184*	*184*	*184*	*184*
Proportion (%)					*78.4*	*60.8*	*32.2*	*13.2*	*5.7*	*2.2*	*1.3*	*0.4*	*0*
